# Structure of Herpes Simplex Virus Glycoprotein D Bound to the Human Receptor Nectin-1

**DOI:** 10.1371/journal.ppat.1002277

**Published:** 2011-09-29

**Authors:** Paolo Di Giovine, Ethan C. Settembre, Arjun K. Bhargava, Micah A. Luftig, Huan Lou, Gary H. Cohen, Roselyn J. Eisenberg, Claude Krummenacher, Andrea Carfi

**Affiliations:** 1 Department of Biochemistry and Molecular Biology, IRBM P. Angeletti, Pomezia, Rome, Italy; 2 Protein Biochemistry, Novartis Vaccine and Diagnostics, Cambridge, Massachusetts, United States of America; 3 Department of Biochemistry, School of Dental Medicine, University of Pennsylvania, Philadelphia, Pennsylvania, United States of America; 4 Department of Microbiology, School of Dental Medicine, University of Pennsylvania, Philadelphia, Pennsylvania, United States of America; 5 Department of Pathobiology, School of Veterinary Medicine, University of Pennsylvania, Philadelphia, Pennsylvania, United States of America; University of Tübingen, Germany

## Abstract

Binding of herpes simplex virus (HSV) glycoprotein D (gD) to a cell surface receptor is required to trigger membrane fusion during entry into host cells. Nectin-1 is a cell adhesion molecule and the main HSV receptor in neurons and epithelial cells. We report the structure of gD bound to nectin-1 determined by x-ray crystallography to 4.0 Å resolution. The structure reveals that the nectin-1 binding site on gD differs from the binding site of the HVEM receptor. A surface on the first Ig-domain of nectin-1, which mediates homophilic interactions of Ig-like cell adhesion molecules, buries an area composed by residues from both the gD N- and C-terminal extensions. Phenylalanine 129, at the tip of the loop connecting β-strands F and G of nectin-1, protrudes into a groove on gD, which is otherwise occupied by C-terminal residues in the unliganded gD and by N-terminal residues in the gD/HVEM complex. Notably, mutation of Phe129 to alanine prevents nectin-1 binding to gD and HSV entry. Together these data are consistent with previous studies showing that gD disrupts the normal nectin-1 homophilic interactions. Furthermore, the structure of the complex supports a model in which gD-receptor binding triggers HSV entry through receptor-mediated displacement of the gD C-terminal region.

**Authors Summary**

Herpes simplex virus (HSV) is a widespread human pathogen. Four viral glycoproteins (gD, gB, gH/gL) are required for HSV entry into host cells. gD binding to a cell surface receptor triggers conformational changes in the other viral glycoproteins leading to membrane fusion and viral entry. Two structurally unrelated cellular protein receptors, nectin-1 and HVEM, can mediate HSV entry upon binding to gD. The structure presented here reveals the molecular basis for the stable interaction between HSV-1 gD and the receptor nectin-1. Comparison with the previously determined structures of the gD/HVEM complex and unliganded gD shows that, despite the fact that the two receptors interact with different binding sites, they both cause a similar conformational change in gD. Therefore, our data point to a conserved mechanism for receptor mediated activation of the HSV entry process. In addition, the gD/Nectin-1 structure reveals that the gD-binding site overlaps with a surface involved in nectin-1 homo-dimerization. This observation explains how gD interferes with the cell adhesion function of nectin-1. Finally, the gD/Nectin-1 complex displays similarities with other viral ligands bound to immunoglobulin-like receptors suggesting a convergent mechanism for receptors selection and usage.

## Introduction

Herpes simplex virus (HSV) enters cells by fusing its envelope with a membrane of the host cell [1]. Five viral envelope glycoproteins participate in the cell entry process. First, glycoprotein C (gC) and gB promote attachment by interacting with cell surface proteoglycans, then gD binds to a specific receptor [2], [3]. The gD-receptor interaction initiates the process that ultimately leads to gB-mediated membrane fusion [4], [5]. Depending on the cell type, fusion occurs at the cell surface or, after endocytosis of virions, with an endosomal membrane in a low pH-dependent or independent manner [6], [7]. Regardless of the entry pathway gD, gB, gH/gL and a cellular gD receptor are required for entry [8].

Several cell surface molecules can bind gD and mediate HSV entry into human cells [1]. The immune modulator HVEM (herpesvirus entry mediator) is a member of the Tumor Necrosis Factor Receptor (TNFR) family and is used by wild type (wt) HSV type 1 (HSV-1) and HSV-2 [9]. HVEM was the first described herpes simplex virus receptor and is expressed at low levels on fibroblasts and on ocular cell types [10], [11], [12]. However, the cell adhesion molecule nectin-1 (HveC, CD111) is the primary receptor for HSV-1 and HSV-2 on neurons, keratinocytes and epithelial cells [12], [13], [14], [15]. In addition, HSV-1 gD binds to heparan sulfate (HS) specifically modified by 3-O-sulfotransferases (3-OS-HS) [16]. Recently it has been shown that the interaction between gD and its receptor is not only required to trigger the HSV fusion machinery but also to direct the virus to the endocytic pathway in some cell types [6], [17], [18], [19], [20].

We previously reported the structure of HSV-1 gD alone and bound to HVEM [21], [22]. The gD ectodomain consists of 316 residues and is formed by a core with a variable-type immunoglobulin fold (IgV, residues 55 to 185) that is wrapped by a N-terminal extension and a C-terminal proline-rich extension. The first 20 N-terminal residues are flexible and extended in the receptor-free form of gD. However, when gD is bound to HVEM these residues fold back to form a hairpin structure that contains all the amino acids contacting this receptor [21], [23]. The first 32 N-terminal residues of gD are also involved in binding to 3-OS-HS but are dispensable for nectin-1 binding and usage [24].

The C-terminal extension of the gD ectodomain past residue 255 is also flexible and its location and structure were determined after stabilization in a disulfide-bonded gD dimer [22]. In this receptor-free structure, the C-terminal region folds back around the core towards the N-terminus and amino acids 280 to 306 fill the space occupied by the N-terminal first 20 residues of gD in the gD/HVEM complex. In this conformation, the C-terminal gD amino acids were positioned directly over several residues previously implicated in nectin-1 binding [22]. Despite the key insights into the gD/Nectin-1 interaction obtained from mutagenesis studies a structural model for the gD/Nectin-1 complex has been missing.

The nectin-1 ectodomain spans residues 31 to 346 forming three domains with an Ig-like fold (V-C1–C2) and contains 8 potential sites for N-linked oligosaccharides [25], [26]. Chimeric receptor molecules were used to show that the binding site for gD involves mainly the N-terminal V-domain of nectin-1 (residues 31–143) [27], [28] and that residues 64–94 in particular are part of the binding epitope [29]. In addition, linear epitopes of two neutralizing monoclonal antibodies directed at nectin-1, CK6 and CK8, were mapped to residues 80–105 [30].

Nectin-1 form *cis*-dimers at the cell surface to interact with nectin-1 or other nectin family members *in trans* (different cells) at cell junctions [31]. The crystal structure of the nectin-1 ectodomain has revealed that the V-domain mediates receptor dimerization [26] and it was proposed that these dimers may be representative of the nectin-1 *cis*-interaction [26]. Importantly, HSV gD has been shown to interfere with nectin-1 mediated cell-adhesion [28], [32], [33].

Given the importance of the gD/Nectin-1 interaction in HSV entry and the need to gain insights into how two structurally different receptors, nectin-1 and HVEM, trigger HSV entry we sought to obtain a molecular view of the interaction between gD and nectin-1. Here we present the structure of the gD/Nectin-1 complex determined by x-ray crystallography to 4.0 Å resolution and accompanying mutagenesis data. The comparison of the new structure with the previously determined gD/HVEM and unliganded gD structures suggests a common mechanism of receptor-induced conformational changes as a trigger of HSV membrane fusion.

## Results

### Structure determination and refinement

**Figure 1:**
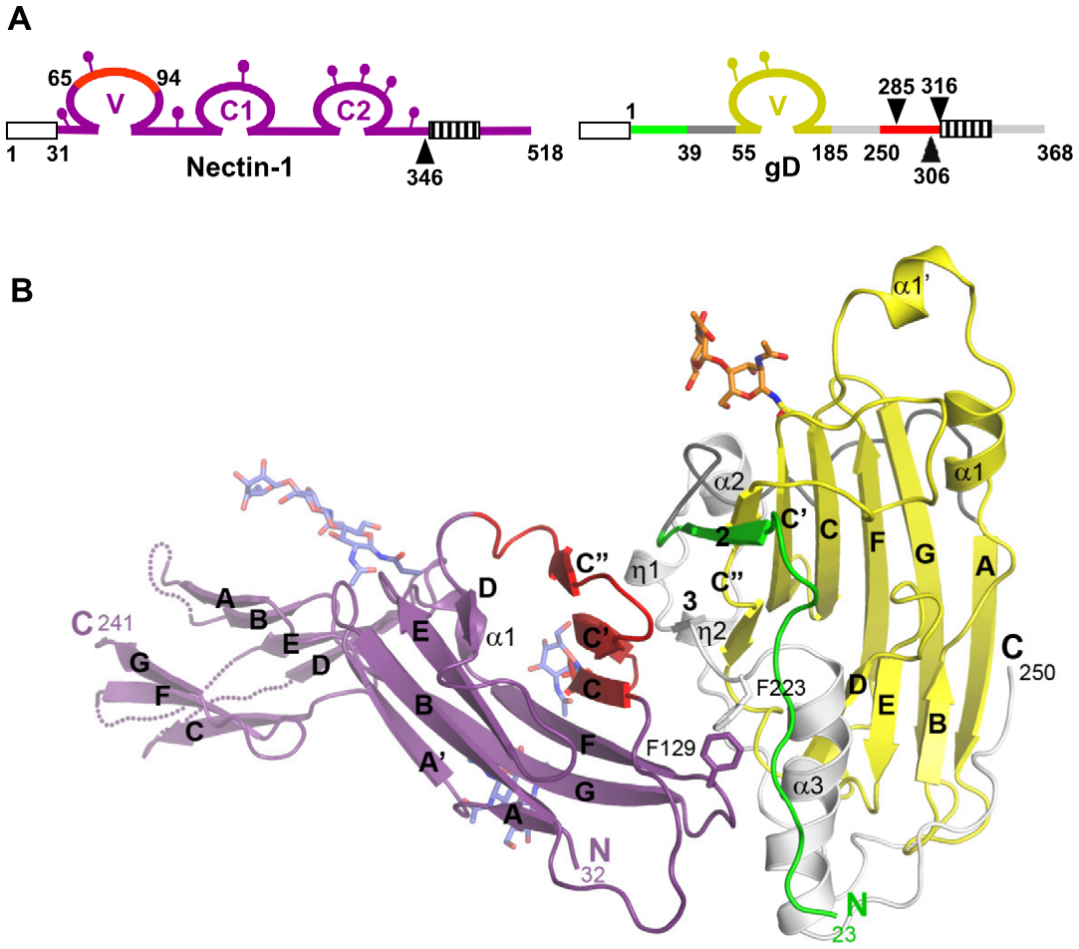
Structure of the gD/Nectin-1 complex. **A**. Schematic representations of human nectin-1 and HSV-1 gD. Signal peptides are shown as white boxes and transmembrane regions are shown as hatched boxes. Lollipops represent N-glycosylation sites. For nectin-1, numbering starts at methionine 1 of the open reading frame while for gD it starts at lysine 1 of the mature glycoprotein. Arrowheads indicate the location of truncations for production of nectin-1(346t), gD285t, gD306t and gD316t. Nectin-1 is colored in violet with a region previously implicated in gD binding colored in red. The gD Ig core is in shown in yellow, residues forming the HVEM binding hairpin are in green, residues 39 to 55 from the N-terminal extension are in dark grey, residues 185 to 250 from the C-terminal extension are in light gray and residues 251 to 316 are in red. **B**. Ribbon representation of the gD/Nectin-1 complex. The color code is the same as in Fig. 1A. The secondary structure elements are labeled as in Carfi *et al.* [21]. The β-strands are labeled according to the Ig V-fold. Unsolved loops in the distal portion of the nectin-1 C1 domain are drawn as dotted lines.

Truncated forms of the gD ectodomain (gD285t, residues 1–285; gD306t, residues 1 to 306; and gD316t, residues 1 to 316) and the full length nectin-1 ectodomain (residues 31–345; **Fig. 1A**) were used for complex formation and crystallization. Crystals were obtained for the gD285t/Nectin-1 complex whereas the longer forms of gD (i.e. gD306t or gD316t) failed to produce crystals. Previous experiments have demonstrated a 20–40 fold decreased affinity for nectin-1 by gD306t (K_D_ = 1.8 µM) compared to the shorter gD285t (gD285; K_D_ = 70 nM) [22]. This decreased affinity and the intrinsic flexibility of C-terminus of the gD ectodomain may have hindered crystallization of the longer forms. In addition, crystals from the gD285t/Nectin-1 complex could not be easily reproduced. The nectin-1 ectodomain contains several consensus N-glycosylation sites (**Fig. 1A**) and heterogeneity in glycosylation may interfere with crystallization [26]. However, attempts to reduce protein heterogeneity by enzymatically deglycosylating nectin-1 produced in insect or mammalian cells did not facilitate crystallization. Ultimately, a 4.0 Å resolution data set was collected and the structure was determined by the molecular replacement method with the program Phaser [34] and refined with Refmac [35] to an R*_free_* of 28.9% and R*_work_* of 26.5% (**Table S1** and **Fig. S1**).

### Overall structure of the gD/Nectin-1 complex

The crystals belong to the P3_2_21 space group and contain three gD/Nectin-1 complexes per asymmetric unit. Two complexes form a dimer around a two-fold non-crystallographic symmetry (NCS) axis (**Fig. S2**) and the third complex forms an equivalent dimer with a crystallographically related molecule. Experiments with purified proteins in solution suggest that these dimers might have formed in the crystals as a result of the high protein concentration and crystal packing interactions and are unlikely to be biologically relevant (see below).

The structure shows a direct interaction between one monomer of gD and one of nectin-1 **(** **Fig. 1B** **)**. No electron density was observed for residues 1 to 22 and 251 to 285. Both regions are known to be flexible and are presumably disordered in the crystals. In the gD285t/HVEM complex the same C-terminal region of gD was disordered [21]. Moreover, deletions within the first 32 residues of gD do not impair nectin-1 binding and HSV entry in nectin-1 expressing cells [36].

The gD binding site is located exclusively within the N-terminal V-domain of nectin-1, validating previous predictions from *in vitro* binding and infection assays [27], [28], [37]. The nectin-1 V-domain major axis forms an angle of almost 90° with the long axis of the gD IgV-core while the C1 domain points away from gD. The elbow angle between the V and C1 domains is the same as that observed in the unliganded form of nectin-1 [26] and differs by approximately 15° from what was observed in the structure of the closely related poliovirus receptor (necl-5, CD155) (**Fig. S3**) [38]. The C2 domain, though present in the crystals of the complex, was not visible in the electron-density maps and is likely to be disordered in the crystals.

### The gD/Nectin-1 interface

Nectin-1 interacts with gD mainly through the side chains of exposed residues. A total of 1665 Å^2^ of surface area, 829 Å^2^ in nectin-1 and 836 Å^2^ in gD, are buried in the complex. This value is within the range observed for other protein/protein complexes and viral glycoprotein/receptor complexes [21], [39], [40], [41]. Nectin-1 contacts gD exclusively with one β-sheet of the V-domain (strands C″C′CFG) and the intervening loops (**Fig. 1B**). This β-sheet forms an extensive interface with residues from the C- and N-terminal extensions of gD. In particular, the upper part of β-strands C″C′ is close to two short gD β-strands (β2, residues 35–38 and β3, residues 219–221) whereas at the bottom part the same strands are in proximity with two short gD helical turns (η1, residues 199–201 and η2, residues 214–217).

**Figure 2:**
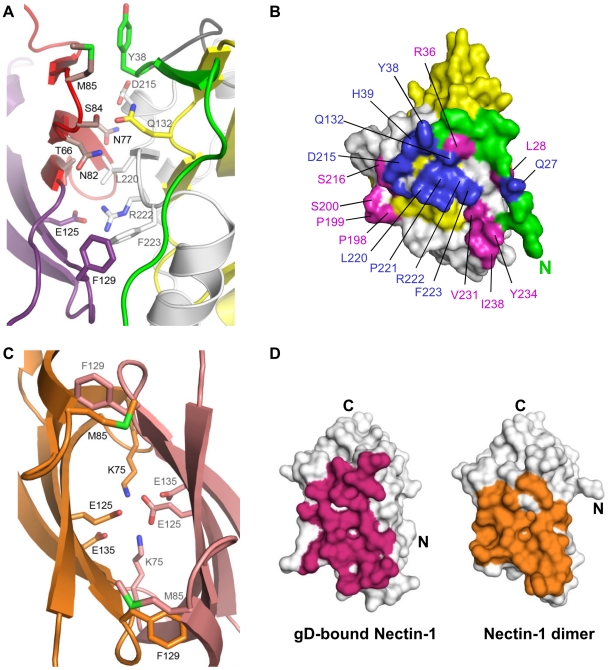
The gD/Nectin-1 and Nectin-1/Nectin-1 interfaces. **A**. Representation of the gD/Nectin-1 interface with key contact residues labeled and displayed in stick representation. Nectin-1 and gD are colored as in Fig. 1. **B**. Surface representation of gD showing the interface with nectin-1. gD is colored as in Fig. 1 with the nectin-1 contact area represented in blue and magenta. Mutations of residues colored blue have been shown to affect nectin-1 binding. Involvement of residues colored magenta has not previously been proposed. Most of the contacts involve residues from the gD C-terminal extension and, Y38 and Q27 from the N-terminal extension. **C**. Representation of a portion of the nectin-1 dimer interface (pdb-id 3ALP). Residues involved in key dimer interactions and that are involved in the gD/Nectin-1 interface are shown in stick representation. One monomer of nectin-1 is colored orange with black labels and the other is in pink with gray labels. **D**. Surface representation of the nectin-1 V-domain highlighting residues involved in either gD binding (magenta, left panel) or nectin-1 dimerization (orange, right panel).

Additional contacts are established by the loop connecting strands F and G of the nectin-1 V-domain. In particular, a prominent interaction involves Phe129, at the tip of the FG loop, which protrudes into a pocket formed by residues from the long α3-helix flanking the IgV-like core of gD and the side chain of Phe223 (**Fig. 1B** and **Fig. 2A, B**). Direct interaction with the gD IgV-core is limited and involves only nectin-1 strand C″ and the side chains of gD Gln132.

### The gD/Nectin-1 structure and previous mutagenesis studies

The structure of the gD/Nectin-1 complex explains the results of previous mutagenesis experiments. On the nectin-1 V-domain, strands C″C′C were predicted to be involved in gD binding based on epitope mapping and chimeric receptor analysis [29], [30], [36], [42] (**Fig. 1B**). In addition, site-directed mutagenesis identified residues important for nectin-1 binding and usage between positions 64 and 94 [29]. Notably, nectin-1 residues Asn77 and Met85, which mutations to alanine hindered HSV entry [43], are now found at the interface with gD (**Fig. 2A**). On gD a number of residues have been shown to affect nectin-1 binding (**Fig. 2A, B**). For instance, gD Tyr38 side chain, a critical residue for nectin-1 binding, is located near the receptor C″ strand and Met85 (**Fig. 2A**). Furthermore, residues Asp215 and Arg222/Phe223 of gD, contact the receptor β-strands C″C′ and the FG loop respectively. The structural data explain the phenotype of a triple mutant of gD, which combined mutations of these residues prevent nectin-1 usage [36], [44]. In particular, the side chain of Arg222 of gD is in salt bridge distance of nectin-1 Glu125 (**Fig. 2A**) consistent with the decrease in gD/Nectin-1 binding affinity seen when Arg222 was mutated to alanine [**45]**, [46****]. Finally, mutation of nectin-1 Phe129 to leucine, a structurally conservative mutation, was found to impair gD binding, although this form of nectin-1 retained some receptor activity [26], [47]. The gD/Nectin-1 structure demonstrates the critical location of this residue at the interface with gD.

### Comparison of the gD/Nectin-1 and Nectin-1/Nectin-1 interface

The structures of the nectin-1 dimer and the gD/Nectin-1 complex reveal similarities between their interfaces. Indeed gD contacts many of the same residues involved in nectin-1 dimerization (**Fig. 2A, C**) and buries a similar surface area on the receptor (1665 Å^2^ vs. 1686 Å^2^) (**Fig. 2D**). In the nectin-1 homo-dimer several hydrogen bonds, two salt bridges (between Lys75 and Glu135), and a number of van der Waals interactions involving Thr63, Phe129 and Met85 are present [26] (**Fig. 2C**). In the complex, both Thr63 and Phe129 of nectin-1 establish van der Waals interactions with Phe233 of gD whereas Met85 is in proximity of gD Tyr38. In addition, in the nectin-1 homodimer two inter-molecular salt bridges between Lys75 and Glu135 are present whereas two Glu125 residues make a potentially unfavorable interaction as they are buried at the dimer interface (**Fig. 2C**). In the complex, the nectin-1 Lys75-Glu135 salt bridge forms intra-molecularly and a new inter-molecular salt bridge is formed between Glu125 of nectin-1 and Arg222 of gD. Therefore, nectin-1 binding to gD leads to the replacement of the potentially unfavorable Glu125-Glu125 interaction at the nectin-1 dimer interface and formation of a new salt bridge (**Fig. 2A, C**).

### Stoichiometry of the gD/Nectin-1 complex and effects of gD binding on nectin-1 homo-dimerization

The β-sheet of the nectin-1 V-domain that contacts gD has been implicated in mediating homophilic and heterophilic trans-interactions in other Ig-superfamily members involved in cell adhesion (e.g. necl-1 [48], CAR [49], JAM [41] and SLAM [50]). The same surface on the nectin-1 V-domain has been shown to mediate its dimerization [26] (**Fig. 2D**). Together these data are consistent with gD binding preventing nectin-1 from interacting with itself as previously suggested by size exclusion chromatography (SEC) experiments [25], [28].

To analyze the stoichiometry of gD/Nectin-1 binding in solution and to gain further insights into the effects of gD binding on nectin-1 oligomerization and homophilic interaction, we combined SEC with multi-angle light scattering analysis (MALS). Differently from SEC, which is dependent on the hydrodynamic radius of molecules, MALS is not affected by the shape of the molecules and provides with the absolute molar mass of the particles measured. The elution volume of the nectin-1 ectodomain (MW 40 kDa) in SEC experiments suggested an apparent MW of 115 kDa, however MALS revealed a MW of 71 kDa (data not shown). Together these data are consistent with nectin-1 forming an elongated dimer in solution as revealed by the crystal structure [26]. MALS of the gD/Nectin-1 complex yielded a MW of 72 kDa in line with the formation of a 1∶1 complex of monomers (data not shown). These analyses are in agreement with previous experiments demonstrating that gD binding prevents functional nectin-1 homo-dimerization and interferes with nectin-1 mediated cell adhesion [32], [33] and support the notion that the gD/Nectin-1 dimers, seen in the crystals, are not formed in solution (**Fig. S2**).

### Mutations of nectin-1 and effect on gD binding

To gain additional insight into the involvement of specific amino acids in the formation of a stable gD/Nectin-1 complex and to assess the relative contribution of the CC′C″ region versus the FG loop, we targeted four nectin-1 residues located at the interface with gD for mutagenesis. We hypothesized that the CC′C″ region would rely less on single residues for binding and that the protruding phenyl ring of Phe129 was critical for function. Thus we selected Thr66, Asn82, Ser84 in the CC′C″ region and Phe129 at the tip of the FG loop (**Fig. 2A**). Ser84 was mutated to tyrosine (S84Y) or arginine (S84R), Asn82 was mutated to a threonine (N82T) and Thr66 to glutamine (T66Q). The changes in the size of the side chains at these positions were designed to destabilize the interaction with gD either by steric obstruction or by interfering with the formation of hydrogen bonds. On the other hand, the structure predicted that mutation of Phe129 to serine (F129S) or alanine (F129A) should prevent formation of favorable hydrophobic contacts and potential stacking interactions. In this context F129W is considered a conservative mutation. Importantly, Asn82, Ser84 and Phe129 are located in exposed positions in nectin-1 and we considered it unlikely that their mutation would affect the folding of the receptor.

**Figure 3:**
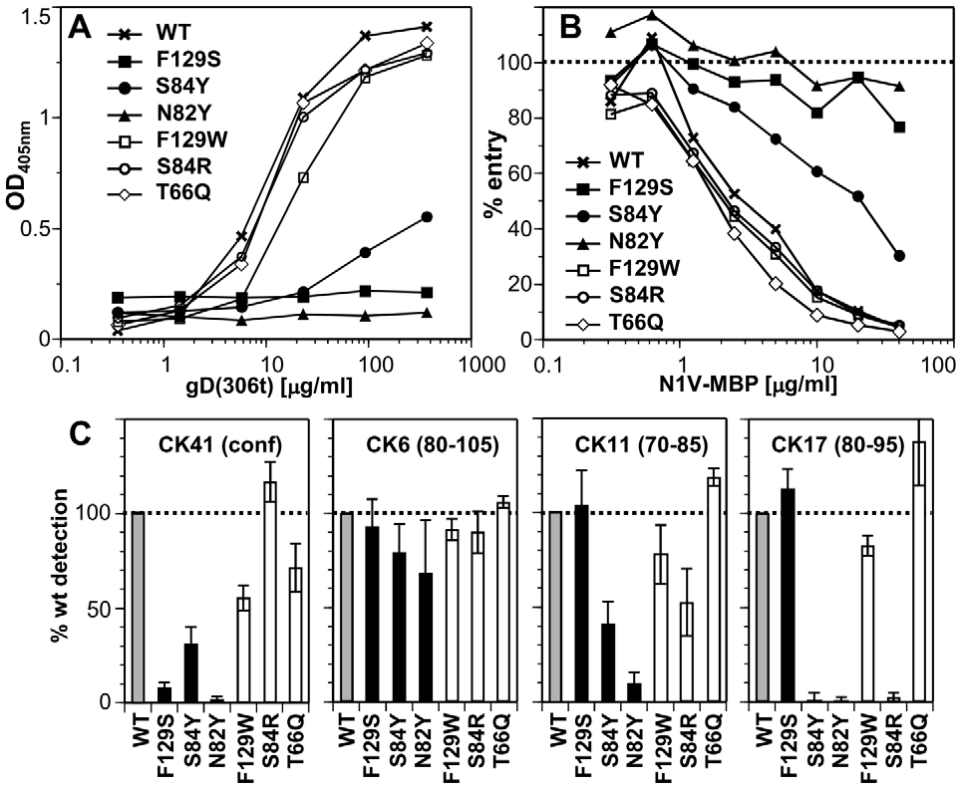
Effect of nectin-1 mutations on binding to gD. **A**. Binding of purified gD(306t) to immobilized N1V-MBP by ELISA. Dilutions of gD were added to the same amount of immobilized N1V-MBP proteins. Bound gD was detected with polyclonal rabbit serum R8. **B**. Blocking of entry of HSV-1 KOS-tk12 into HeLa cells with soluble N1V-MBP proteins. Virus was preincubated with various concentrations of the indicated purified proteins for 1h and then added to HeLa cells. Entry was monitored 6h post infection by measuring β-galactosidase activity. The results are reported as percent of β-galactosidase signal compared to infection in the absence of soluble inhibitor (dotted line). A representative experiment is shown. **C**. Antigenic characterization of N1V-MBP mutants by ELISA. The purified wt fusion protein and each mutants were captured by the indicated immobilized anti-nectin-1 Ig and detected with anti-MBP antibody conjugated to HRP. Detection of each mutant is represented as percent of wt detection. An average of at least 5 experiments is shown with standard deviation.

To analyze the effects of these mutations on binding of nectin-1 to gD *in vitro*, we produced chimeras comprising the nectin-1 V-domains (aa 31–146) fused at its C-terminus to the maltose binding protein (N1V-MBP). We first analyzed the ability of the N1V-MBP proteins to bind gD by ELISA (**Fig. 3A**). While S84R and T66Q nectin-1 mutants bound to gD(285t) similarly to wt, S84Y and N82Y mutation markedly decreased binding. Furthermore, the aromatic side chain at Phe129 was critical for gD complex formation as F129S mutation severely compromised gD binding while F129W did not (**Fig. 3A**).

We next analyzed the ability of mutant N1V-MBP proteins to interact with gD on virions. We tested whether the purified soluble receptors blocked HSV entry into cells by competing with cell surface nectin-1. We used HeLa cells as targets since soluble nectin-1 V-domain produced in baculovirus efficiently blocked entry in these cells [30]. Consistent with the ELISA results, proteins with the mutations F129S or N82Y failed to block entry (**Fig. 3B**). Mutation S84Y had intermediate blocking activity, which correlated with its reduced binding to gD. The other mutants (F129W, S84R and T66Q), which bound gD like the wt protein by ELISA, retained the ability to block virus entry. These observations indicates that these mutants bind virion gD with sufficient affinity to compete with cell surface nectin-1 for binding to gD on the viral envelope.

### Nectin-1 mutations and CK41 MAb binding

We determined the effects of nectin-1 mutations on binding to the conformation-dependent MAb CK41. This antibody blocks binding of nectin-1 to gD and efficiently prevents nectin-1 usage for HSV entry [30]. We therefore anticipated that mutations designed to prevent gD binding would also affect the interaction with CK41. Indeed mutations that affected gD binding (i.e. S84Y, N82Y and F129S) also hindered CK41 binding (**Fig. 3C**). In addition, since the conserved mutation F129W decreased CK41 recognition by 50%, without affecting gD binding, we infer that Phe129 is likely part of the CK41 epitope. Change of Ser84 to arginine does not affect CK41 detection, while a change to tyrosine decreased binding of this MAb. Overall these data mirror the binding properties of these nectin-1 mutants to gD and are consistent with the prediction that the CK41 epitope involves residues critical for gD binding [30].

### Mutations of nectin-1 affect its function as an HSV receptor

**Figure 4:**
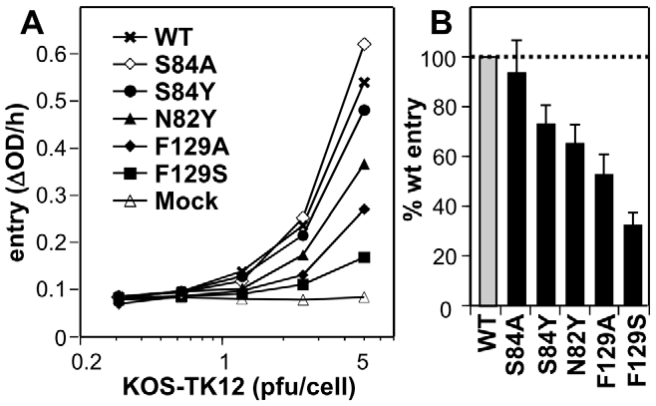
Effect of nectin-1 mutations on HSV entry. A . Receptor negative B78H1 cells were transfected with full-length nectin-1 carrying the indicated mutations. HSV KOS-tk12 virus was added at various MOI. Entry was recorded 6h post infection by measuring β-galactosidase activity. A representative experiment is shown. Background signal from mock-transfected cells is also shown. **B**. Summary of all the entry data at an MOI of 5 pfu/cell. Levels of entry into cells expressing nectin-1 mutants are reported as the percentage of entry into B78H1 cells transfected with wt nectin-1. An average of 6 experiments is shown with standard deviation.

To further assess the functional role of individual residues at the gD/Nectin-1 interface, we tested a subset of mutants in a viral entry assay. Mutations were engineered in full-length nectin-1 and transfected into receptor-negative B78H1 mouse melanoma cells [32]. Upon transient expression of wt nectin-1, B78H1 cells become susceptible to HSV entry (**Fig. 4A**) [32]. While all tested mutants allowed some level entry of HSV-1 KOS tk12 in a dose dependent manner, mutants F129S and F129A exhibited a marked decrease in entry compared to wild type (**Fig. 4B**). These results are consistent with the reduced binding affinity of the mutated receptor to gD. Nectin-1 mutants N82Y and S84Y were only partially reduced in their ability to support HSV entry despite exhibiting profound decrease in N1V-MBP binding to gD by ELISA. Such a discrepancy between binding and entry function has been previously observed [47], [51]. It is likely that the decrease in affinity observed between the purified proteins is less critical in the context of the cell-virus interaction due to avidity effects (i.e. HSV binding to multiple receptor molecules and to cell surface HS) thus making the entry assays more permissive [47], [51]. Overall, however, the phenotypes of the mutants agreed with their importance for stabilizing the interface indicated by the structure of the complex.

### Nectin-1 and HVEM binding sites on gD are mutually exclusive and require the displacement of gD C-terminal region

**Figure 5:**
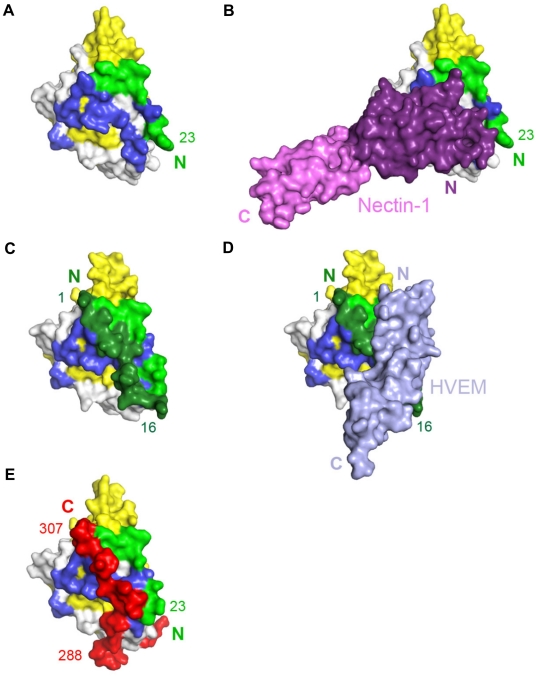
Unliganded gD and gD/receptor complexes. **A, B**. Surface representation of gD bound to nectin-1, with nectin-1 removed (A) or present (B). Residues 23–38 of gD are colored light green, the V-domain (residues 56–185) is colored yellow and the other residues from N-terminal (residues 39–55) and C-terminal (residues 186–250) extensions are in gray. Residues buried by nectin-1 in the gD/Nectin-1 complex are colored in dark blue. The V domain of nectin-1 is colored violet and the C1 domain is pink. **C, D**. View of gD bound to HVEM with HVEM removed (C) or present (D). Colored as in panel A with the N-terminal region involved in HVEM binding colored in dark green (residues 1 to 16) and in light-green (residues 17–38). Residues 1–16 of gD, which were not localized in the gD/Nectin-1 structure, fold back to form a hairpin structure when HVEM is bound. In the latter conformation, these residues mask the nectin-1 binding site. HVEM is shown in light blue. **E**. Conformation of unliganded gD colored as in panel A with residues 260–306 from the C-terminal extension colored in red.

Comparison of the gD/Nectin-1 and gD/HVEM structures reveals that the two receptors bind to different but overlapping sites. Although each receptor buries a comparable area on gD, for the most part their binding sites do not involve common residues. In fact, the binding site of HVEM is located exclusively within the first 32 N-terminal residues of gD that are folded in a hairpin-like structure [21]. The same hairpin does not form in the nectin-1 complex, rather the first N-terminal 22 residues of gD point towards the solvent (**Fig. 5A**–**D**). However, a large portion of the gD surface contacted by nectin-1 would be covered by these N-terminal residues if they were adopting the same hairpin structure observed in the gD/HVEM complex (**Fig. 5A**–**D**). Thus the structural comparison reveals that nectin-1 and HVEM bind to different sites on gD but the binding of one receptor should prevent the binding of the other.

The unliganded gD structure showed the C-terminal residues 268–306 wrapped around the gD core (**Fig. 5E**) [21], [22]. Notably, in this position, these residues cover a large portion of the nectin-1 binding site (**Fig. 5A, B and E**). Therefore, formation of the gD/Nectin-1 complex requires disruption of the contacts between the core and the C-terminus of the gD ectodomain [22], [52]. Interestingly, this separation is also required for the formation of the HVEM-binding N-terminal hairpin of gD (**Fig. 5C and E**) [22].

## Discussion

The structure and mutagenesis data reported here provide the molecular basis for the interaction between gD and its cellular receptor human nectin-1. The structure shows the key features of the interface between the nectin-1 V-domain and the core of gD [28]. The binding site for gD overlaps with a nectin-1 dimerization interface explaining how gD interferes with the cell-adhesion function of this receptor. In addition, the gD/Nectin-1 complex reveals structural similarities with other viral ligands bound to receptors with an Ig-like fold, thus pointing to a convergent mechanism for receptor selection and usage by otherwise unrelated viruses. Importantly, comparison of the gD/Nectin-1 structure with the previously determined gD/HVEM structure indicates that despite contacting different amino acids the two receptors compete for binding to gD. Finally, a comparison between the two gD-receptor structures and the unliganded gD structure provides additional insights into the mechanism of receptor-mediated activation of the HSV fusion machinery.

### The gD/Nectin-1 interface

The gD/Nectin-1 structure confirms the presence at the interface of a number of gD residues that were previously identified by mutagenesis to be important for nectin-1 usage and HSV entry [36], [44], [46], [53]. gD contacts a large surface on the nectin-1 V-domain composed mainly by residues from the C″C′CFG β-sheet. In other cell adhesion molecules of the Ig-superfamily this same region is involved in homophilic and heterophilic trans-interactions and nectin-1 uses the same surface to homo-dimerize [26]. Therefore, and in agreement with our SEC/MALS data, formation of the gD/Nectin-1 complex requires dissociation of the nectin-1 dimers. Consistent with these findings, gD can prevent nectin-1 mediated cell aggregation [32] and HSV infection is favored by prior disruption of cell junctions [54].

**Figure 6:**
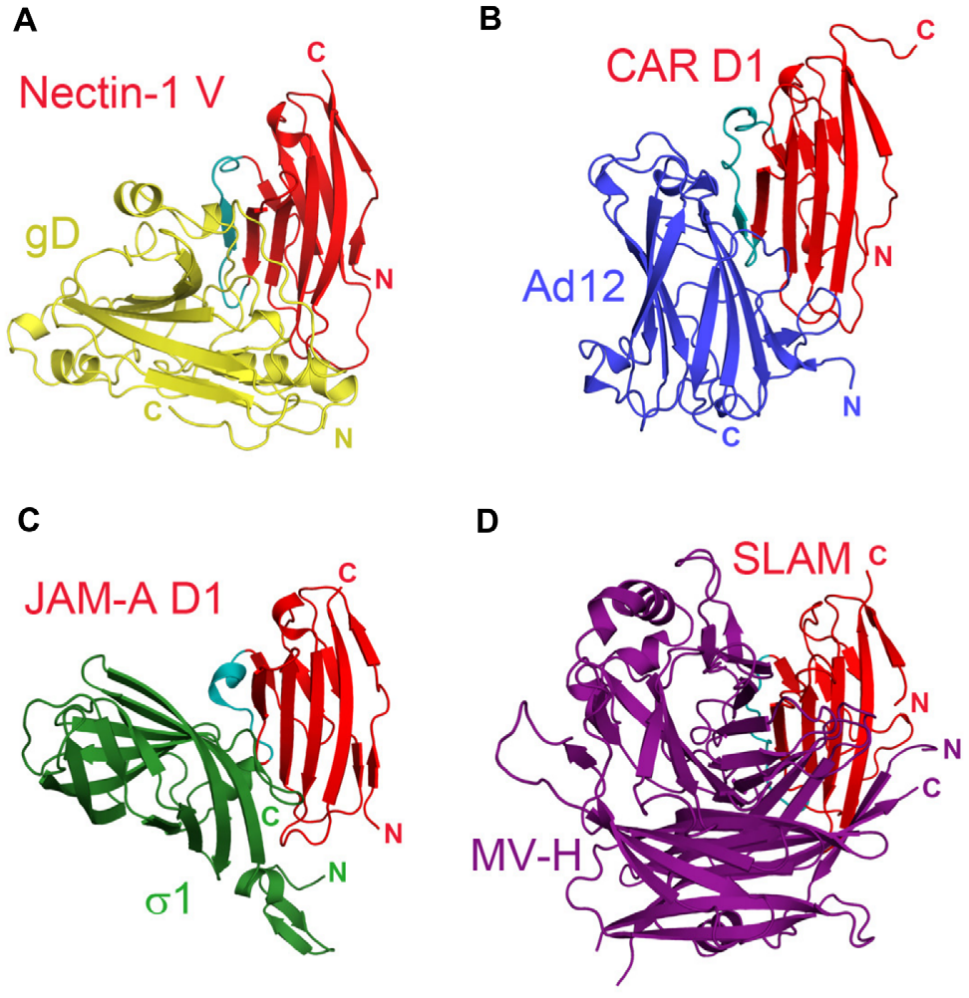
Similarities in the interactions between adhesion molecules with Ig-like fold and viral receptor-binding proteins. The Ig-like domains of the receptors are shown in similar orientation and colored in red with the region that appears most structurally variable in cyan. **A**. Nectin-1 V-domain bound to HSV gD (gold). **B**. Coxsackie and Adenovirus receptor, CAR, domain D1 bound to Ad12 of adenovirus (blue) (pdb-id 1KAC). **C**. Junctional adhesin molecule-A, JAM-A, domain D1 bound to reovirus σ1 (green) (pdb-id 3EOY). **D**. Signaling lymphocyte activation molecule, SLAM, bound to measles virus hemagglutinin, MV-H, (purple) (pdb-id 3ALX).

Notably, non-enveloped adenovirus, reovirus and measles virus bind to a similar epitope on their respective receptors, the Ig-like cell-adhesion receptors CAR, JAM-A and SLAM (**Fig. 6A**–**D**) [50], [55], [56], [57]). Similarly to HSV, these viruses disrupt the homophilic trans-interactions of their receptors [41], [49]. Binding to cell-adhesion molecules may ultimately favor release of these viruses by opening intercellular junctions [58].

### Contribution of nectin-1 phenylalanine 129 to gD binding

**Figure 7:**
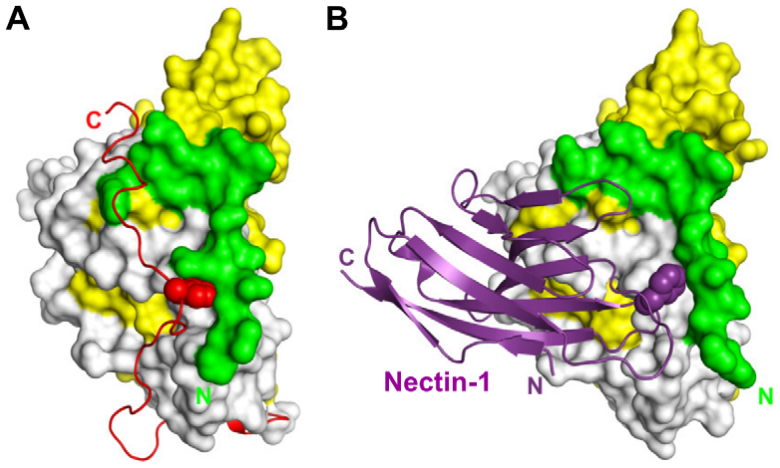
Functional pocket on the surface of gD. Comparison between unliganded gD and gD/Nectin-1 complex. gD N-terminal residues 23–38 are green, the Ig-like V-domain is colored yellow (aa 56–185), the C-terminal region (aa 268–306) is shown in red and the remaining residues are white. **A**. In unliganded gD, the C-terminus of the ectodomain (red) is maintained in place by the insertion of Trp294 into a pocket made of the gD N-terminal residues, the α3-helix and residues from the Ig-core. This interaction is critical for function. **B**. The side chain of Phe129 (purple) located on the FG loop of the nectin-1 V-domain protrudes into the same pocket in the complex with gD. This binding configuration is not compatible with the native position of the gD C-terminus.

On the nectin-1 side, point mutations of residues in the C″C′C region can affect binding to gD (e.g. S84Y, N82Y); however, they have limited effects on nectin-1 as a mediator of HSV entry. This suggests that the interactions established by single amino acids in this region may not be critical for function. Indeed, the same conclusion can be drawn from the gD side where multiple mutations are needed to abolish nectin-1 usage [36], [44]. This is quite different from the role of Phe129 at the tip of the FG loop of the nectin-1 V-domain. Mutations of Phe129 to alanine showed that this residue plays an important role for tight gD binding and for HSV entry. Of note, Phe129 protrudes into a pocket on the gD surface occupied in the unliganded gD by the C-terminal residue Trp294 (**Fig. 7**). Therefore, nectin-1 Phe129 effectively substitutes for gD Trp294 and provides a key contribution to the stable displacement of the gD C-terminal region.

Importantly, Phe129 is conserved in the poliovirus receptor necl-5 and nectin-2, consistent with the finding that a chimera composed of the necl-5 or nectin-2 V-domain where strands C″C′ were replaced with the corresponding residues of nectin-1 retains binding to gD and allows HSV infection [29], [59]. The equivalent FG loop in necl-5 has been shown to be at the interface with poliovirus and to be important for poliovirus binding [38], pointing to a conserved feature in the interaction between HSV and poliovirus with their respective receptors.

### Comparison with the gD/HVEM complex and implication for gD activation

Nectin-1 and HVEM are two gD receptors that belong to different structural families. Consistently, the gD/Nectin-1 complex shares only limited similarity with the gD/HVEM complex **(** **Fig. 5**). The binding site of nectin-1 on gD differs dramatically from that of HVEM. The latter contacts only residues within the first N-terminal 32 residues of gD folded in a hairpin-like structure and the interaction involves several hydrogen bonds through main and side chain atoms [21]. The nectin-1 V-domain, instead, contacts a large surface on gD formed mostly by residues from the C-terminal extension and some amino acids from the N-terminal region. However, structural superposition of the two gD-receptor structures reveals that most of the residues involved in nectin-1 binding are buried by the gD-N-terminal residues in the gD/HVEM complex (**Fig. 5**). Thus, despite their considerably different binding sites, each receptor is likely to interfere with binding of the other. Indeed soluble nectin-1 can block virus entry in HVEM expressing cells [60].

A comparison between the gD-receptor complexes and the structure of unliganded gD provides additional insights in the mechanism of receptor-mediated activation of gD. In the absence of receptors residues from the gD C-terminal region (residues 285–306) are anchored by Trp294 on the core of gD. In this conformation, the C-terminus of gD occludes the nectin-1 binding site and fills the space occupied by gD N-terminal residues in the gD/HVEM complex. This is consistent with the increase receptor affinity of the C-terminally truncated form of gD used in this study compared to the full length molecule [25], [61]. Therefore, for both receptors complex formation requires the displacement of residues from the gD C-terminal region.

### Role of the C-terminus of the gD ectodomain

During HSV entry, gD interacts with gH/gL and possibly gB [4], [62], [63] but how gD triggers conformational changes in the other glycoproteins remains unclear. Several observations point to a key role of the gD C-terminal region. A soluble gD molecule encompassing the entire ectodomain allows entry of a gDnull virus (i.e. devoid of envelope gD) in receptor expressing cells, whereas a truncated form of gD lacking residues 260–316 does not [64]. Moreover, mutagenesis data showed that the C-terminal region is essential for virus entry [52], [62], [64], [65], [66]. These data support a role for the gD C-terminal region in activation of the fusion/entry process. They also suggest a critical role for this region for the interaction with other viral glycoproteins. Exposure of the C-terminal region upon receptor binding therefore provides a timely and cell specific trigger for the activation of the HSV entry process. The conformation of the C-terminus in receptor-bound gD has not been determined due to its high flexibility.

Remarkably, gD has been engineered to bind alternate receptors [67], [68], [69], [70]. Heterologous ligands such as uPA, IL13, and a single chain antibody to HER-2 were engineered in at least three different regions in the gD-N-terminus or replaced the entire gD Ig core. All these ligands are able to mediate entry of HSV virions carrying the chimeric forms of gD in cells expressing the respective receptors. In the absence of structural data on such chimeras, one can only speculate on their mechanism of action during entry. Even in the most extreme recombinant [70], the N-terminal and C-terminal extensions to the Ig core are maintained for activity [71]. Thus, these regions likely contain the necessary sites for binding and activation of the other viral glycoproteins. In the engineered gDs the C-terminus may be exposed upon exogenous receptor binding in a way that is very similar to the model supported by our studies for wt HSV. Alternatively, this functional region may already be exposed in these molecules so that exogenous receptor binding would solely allow the close proximity of viral and cell membranes. In such a situation, expressing a pre-activated gD may lead to a decrease in viral fitness by diminishing the selectivity for target cells. It is likely that HSV evolved to unmask the gD C-terminus only upon receptor binding to ensure efficient tropism in the host. Of note, the initial analysis of the structural organization of gD revealed that the Ig-core acts as structural support to the functional C- and N- terminal regions and suggested that it inserted in an ancestor molecule formed by the N- and C- terminal extensions [21]. The results obtained with the above engineered gDs are consistent with such hypothesis.

In summary, our data reveal the molecular basis for the gD/Nectin-1 interaction. This new structure shows how nectin-1 and HVEM, albeit belonging to different structural families and establishing different interactions with gD, similarly cause the disruption of intra-molecular contacts between the gD C-terminal region and the rest of the molecule thus leading to activation of the entry process through a conserved mechanism. The structure of the gD/Nectin-1 complex also provides a frame to design of inhibitors that would block HSV entry and infection.

## Materials and Methods

### Complex purification

Production of gD-1 KOS (residues 1 to 285, gD(285t)) and human nectin-1 (residues 31–346, gD(346t)) using recombinant baculoviruses and their purification were described previously [21], [25]. The complex used for crystallization experiments was formed by mixing purified gD and nectin-1 in a 1.3∶1 molar ratio. Unbound gD in excess was removed by size exclusion chromatography (SEC) on a analytical Superdex-200 column in 20mM TRIS-HCl pH 8.0 buffer and 300mM NaCl. The complex, which eluted as single peak from the SEC step, was concentrated to 3.5mg/ml and stored at 4°C prior to crystallization.

### Crystallization and data processing

The gD/Nectin-1 complex was crystallized by the vapor diffusion method with 1.0M Na_2_HPO_4_/KH_2_PO_4_ pH 7.2 and 300mM NH_4_SO_4_ as precipitating agents. SDS gels and N-terminal sequencing on washed crystals confirmed the presence of the full-sized proteins. The crystals had a very low reproducibility, suffered from strong anisotropy and the great majority diffracted only up to 7–8 Å resolution at different x-ray synchrotron sources. Nevertheless, after screening more than 100 crystals a 4.0 Å data set was collected at the BM14 beam line at the Advanced Photon Source (APS), Argonne National Lab. (**Table S1**). The HKL suite was used for data integration and scaling [72] and the CCP4 suite was used for further data processing and analysis [73].

Data integration suggested that the crystals belong to the hexagonal P6_2_22 space group, however analysis of the cumulative intensity distribution [73] hinted at the presence of merohedral twinning and as a consequence to a lower symmetry space group. Data analysis with the Merohedral Crystal Twinning server for data integrated in P321 or P312 pointed to the presence of nearly perfect merohedral twinning (α = 0.49) [74]. Due to the difficulty of obtaining similar quality diffraction this data set was used for subsequent structure determination and refinement despite integration in the trigonal space groups resulted in low redundancy (**Table S1**).

### Structure determination and refinement

The structure was determined by the Molecular Replacement (MR) method with the program Phaser [34]. The initial search was carried out in all trigonal and hexagonal space groups with a gD model (PDB entry 2C36: amino acids 27–250) and using all data between 12 and 4.0Å. A clear solution for 3 gD molecules was identified in P3_2_21 whereas no solutions were found in all the other space groups tested. A polyalanine version of the major structural protein of peripheral nerve myelin (1NEU; 27% sequence identity with the V-domain of nectin-1), the C1 domain of the poliovirus receptor necl–5/CD155 (3EOW; 34% identity with the C1 domain of nectin-1) and the perlecan IG3 domain (1GL4 25% identity), after removal of some of the loops, were used as search models for the V, C1 and C2 domains of the receptor, respectively. Keeping the 3 gD molecules fixed, three solutions for the V domain and then for the C1 domains of nectin-1, consistent with 3 gD-nectin-1 complexes in the asymmetric unit, were clearly identified. In addition to the improvements in TFZ, LLG, R_work_ and R_free_ during all the steps of MR several pieces of evidence supported the correctness of the solution. First, the nectin-1 V-domain was positioned in proximity of gD residues previously implicated in nectin-1 binding; second, the N- and C-termini of the C1 and V-domain, respectively, were at a distance from each other compatible with the number of missing residues connecting the two domains; and third the model agreed with considerations on packing and symmetry relation between the molecules in the crystal (see also Results). However, only a poorly contrasted solution for one of the C2 domains could be identified by MR. Despite this solution positioned the C2 domain in proximity of a C1 domain with reasonable crystal contacts, its addition to the model did not improve the R*_free_* or the quality of the electron density maps and therefore was excluded from the refinement.

Simulated annealing composite 2mFo-DFc and mFo-DFc omit electron density maps followed by local three fold NCS averaging for each of the nectin-1 domains were calculated with the program Phenix [75] and used during the initial stages of model building. The resulting electron density maps clearly revealed the location of some of the loops in the nectin-1 V-domain and some of the glycans both of which had been excluded from the MR model (**Fig. S1**). The electron density of the glycans and of residues with large side chains together with the location of disulfide bonds in V and C1 Ig domains were used to confirm the register of the polypeptide chain. The model was refined with Refmac using the ″detwin″ option, overall B-factor refinement and tight non-crystallographic symmetry (NCS) restraints. The twinning fraction estimated by Refmac was of 0.49, in agreement with what reported by the Merohedral Crystal Twinning Server. When the structure was refined without allowing for detwinning of the data the R_free_ was ∼10% higher. Following the initial building the crystal structure of nectin-1 became available [26] and was used to modify the V and C1 domains. Additional refinement showed that although most of the structure remained the same between the two models, modest differences were present in between the structures in some of the V1 loops and residues involved in the gD-nectin-1 interface. The final model includes the full V1 domain but does not include some of the loops in the distal part of the nectin-1 C1 domain which did not have interpretable electron density.

### Production of nectin-1 V-domain-MBP

A DNA fragment corresponding to the variable immunoglobulin domain (V-type) of nectin-1 was amplified by PCR from a plasmid containing the cDNA form of the full length protein (pCK451) [76], using primers designed to insert a stop codon after residue 146. This fragment was inserted into the bacterial gene fusion vector pMAL-p (New England Biolabs) by a blunt-ended ligation into BamH1 restriction site of the plasmid polylinker. This construct produces a fusion with the maltose-binding protein under the control of the Lac repressor that is exported to the periplasm. Mutants were produced using the Quikchange mutagenesis kit (Stratagene). Correctness of all the constructs was verified by sequencing. For protein purification, E. coli BL21 (DE3*) cells were transformed with the constructs, grown at 37°C in LB to an optical density of 0.7 at 600nm and induced with ITPG for 16 h at 23°C. After an overnight culture cells were resuspended in 40mM TRIS pH 7.5, 300mM NaCl, 10% glycerol, 0.03% β-octyl-glucoside (Buffer A, 100ml/liter of culture) and lysed with a microfluidizer. Debris were removed by centrifugation (13000 rpm) for 45 min at 4°C and the supernatant was incubated with amylose resin (New England Biolabs). After binding, the resin was washed with Buffer A and proteins were eluted with 20mM maltose in Buffer A. Proteins were concentrated and purified by size exclusion chromatography on a Superdex S200 column equilibrated with 40mM Tris pH 8.0 and 150mM NaCl. Fractions containing non-aggregated nectin-1 V-domain MBP fusion protein (N1V-MBP) were pooled, concentrated and dialyzed against PBS. All the N1V-MBP mutants were purified similarly to the wild type protein.

### Nectin-1 mutants and gD binding

Purified N1V-MBP fusion proteins were immobilized on a 96 well plate (10 µg/ml in PBS) overnight at 4°C. Plates were blocked with PBS containing 0.05%, Tween20 and 5% nonfat milk (PBS-T milk). Purified gD(306t) (residues 1 to 306) was serially diluted in PBS-T milk and added to the plate [25]. After incubation and washing with PBS-T, bound gD was detected with polyclonal rabbit serum R8 (diluted 1∶500 in PBS-T-milk) and goat anti-rabbit-HRP secondary antibody (KPL Inc). Absorbance at 405 nm was read after addition of ABTS as substrate.

### Blocking HSV infection

HSV-1 KOStk12 [9] was diluted in cell culture medium and pre-incubated with purified N1V-MBP proteins at various concentrations for 1 h at 37°C. The mixture was added to confluent HeLa cell cultures in a 96 well plate (5×10^4^ cells/well) and directly incubated at 37°C for 5–6 h. Lysis and reading of β-galactosidase activity was performed as described below for entry assays.

### CK41 detection by ELISA

Purified anti-nectin-1 monoclonal antibody CK41 [30] was immobilized on an ELISA plate in PBS (10 µg/ml). After blocking with PBS-T-milk, purified N1V-MBP (20 µg/ml) was added in PBS-T-milk for 2 h at RT. The wells were washed and N1V-MBP was detected with HRP-conjugated anti-MBP antibody MBP-17 (Sigma-Aldrich), followed by goat anti-rabbit-HRP secondary antibody (KPL Inc). Absorbance at 405 nm was read after addition of ABTS as substrate.

### Nectin-1 mutants and HSV entry

Mutations were engineered in full-length nectin-1 using the Quikchange mutagenesis kit in plasmid pCK452 expressing full-length human nectin-1α [76]. Plasmids were transfected into B78H1 cells using either Geneporter (2 µg plasmid/ well of subconfluent cells in 6 well plates) or the AMAXA system kit V, 2 µg/10^6^ cells. One day post transfection cells were seeded in 96 well plates (5×10^4^ cells/well). After an overnight culture, cells were infected with dilutions of sucrose-purified HSV-1 KOS tk12. After 6 h incubation at 37°C cells were lysed by adding NP-40 to a final concentration of 0.5%. A 50 µl volume of lysate was mixed with an equal volume of β-galactosidase substrate (chlorophenol red-β-D-galactopyranoside; Roche) and absorbance was read at 595nm for 50 min to record enzymatic activity. Surface expression of nectin-1 mutants was assessed by CELISA using MAb CK6 [30] and was comparable between mutants and wt nectin-1 in transiently transfected B78H1 cells.

### Accession number for genes and proteins mentioned in the text

*Nectin-1*

Swissprot/UniProt: Q15223

NCBI: NP_002846

*HVEM*

Swissprot/UniProt: Q92956

NCBI: AAQ89238

*gD KOS*

Swissprot/UniProt: A1Z0Q5

## Supporting Information

Figure S1 Representative electron density (gray) from a three-fold averaged composite anneal omit map of the nectin-1 V-domain. A region of the final model is shown in the density in stick representation highlighting the presence of two glycosylation sites.
(TIF)

Figure S2 View of a crystallographic dimer of gD/Nectin-1. gD is shown in yellow and orange and nectin-1 is shown in purple and pink.
(TIF)

Figure S3 Comparison of the elbow angle of necl-5 (cyan) and nectin-1 (purple). An approximate difference of 15 degrees exists between the two structures. Loops not built in the nectin-1 model are shown as a dotted line.
(TIF)

Table S1 Data collection and refinement statistics.
(DOC)
