# Supplementary figures and images for: Structure of Herpes Simplex Virus Glycoprotein D Bound to the Human Receptor Nectin-1
Source: PLoS Pathog. 2011 Sep 29;7(9):e1002277. doi: 10.1371/journal.ppat.1002277 (PMC3182920; doi:10.1371/journal.ppat.1002277)

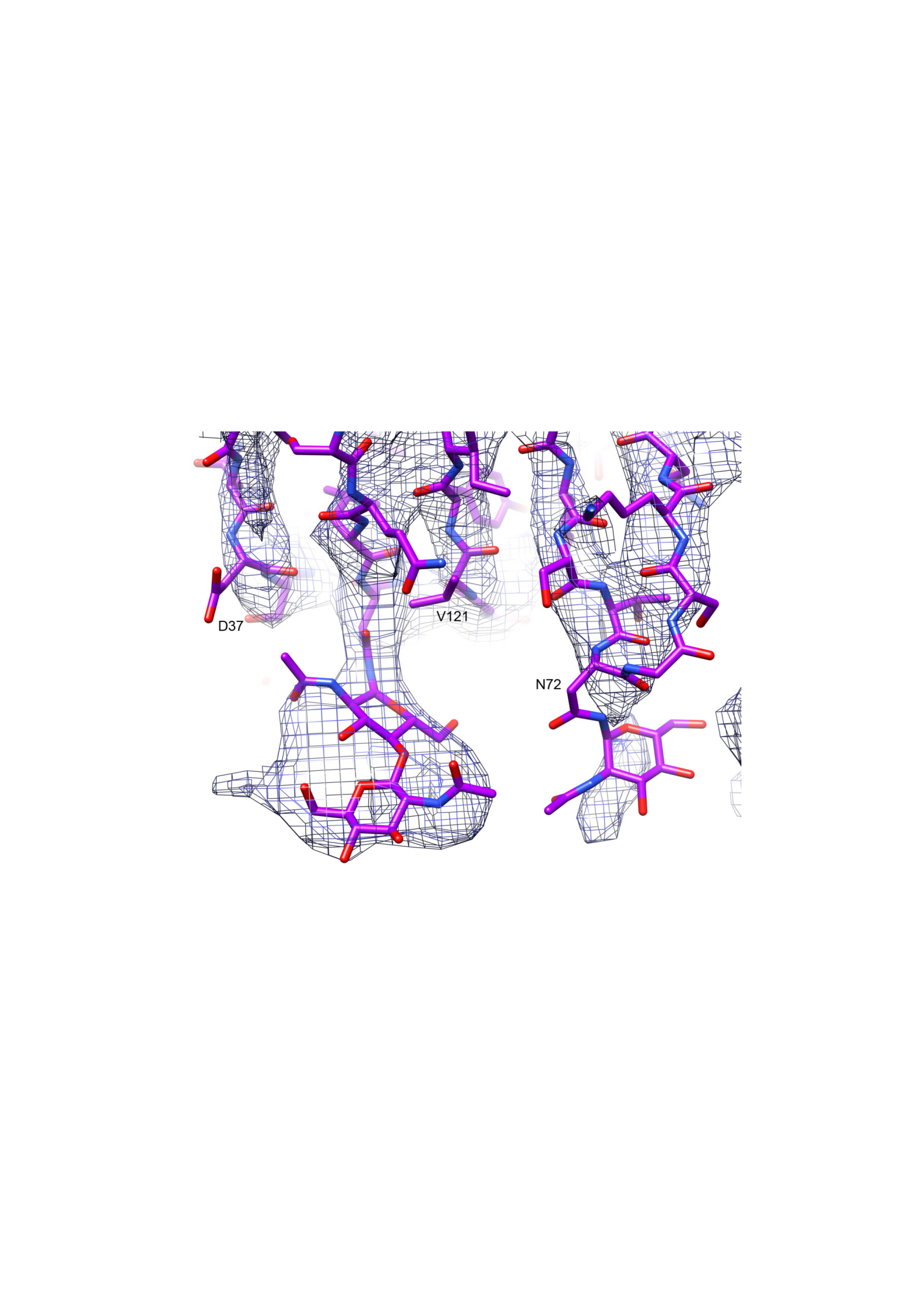

Supplement: Figure S1 — Representative electron density (gray) from a three-fold averaged composite anneal omit map of the nectin-1 V-domain. A region of the final model is shown in the density in stick representation highlighting the presence of two glycosylation sites. (TIF) [file ppat.1002277.s001.tif]

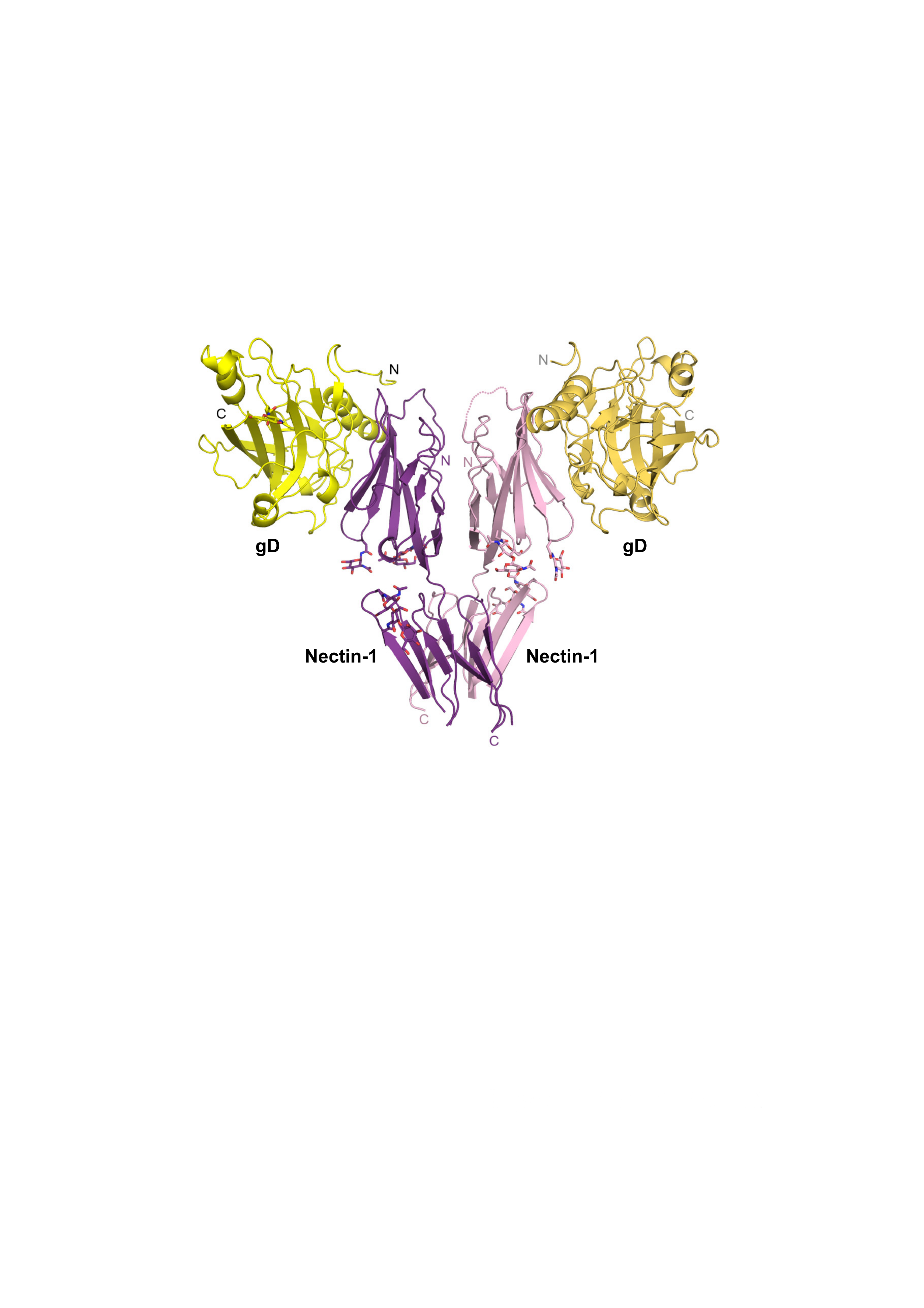

Supplement: Figure S2 — View of a crystallographic dimer of gD/Nectin-1. gD is shown in yellow and orange and nectin-1 is shown in purple and pink. (TIF) [file ppat.1002277.s002.tif]

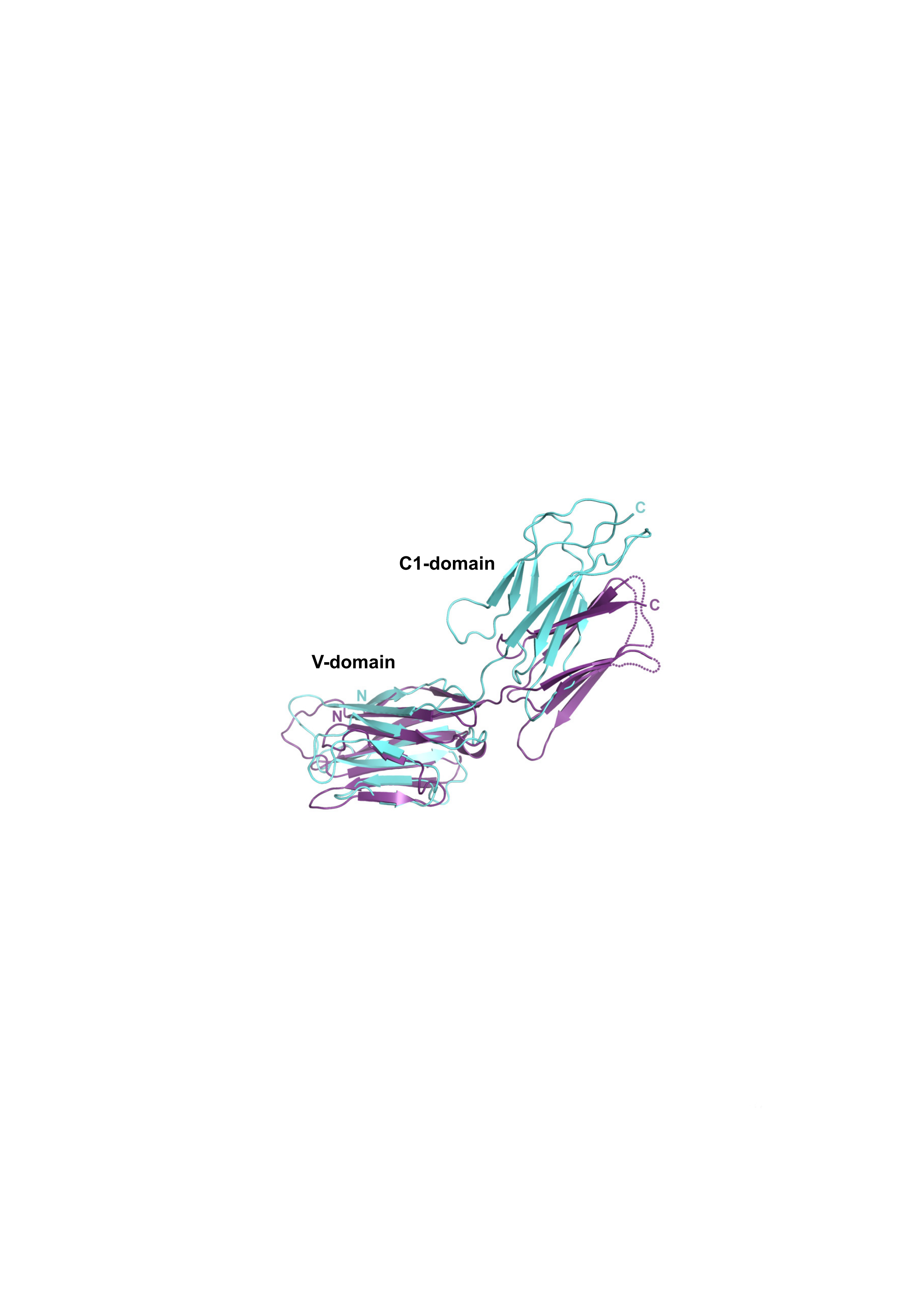

Supplement: Figure S3 — Comparison of the elbow angle of necl-5 (cyan) and nectin-1 (purple). An approximate difference of 15 degrees exists between the two structures. Loops not built in the nectin-1 model are shown as a dotted line. (TIF) [file ppat.1002277.s003.tif]
