# Supplementary material for: Structure of Herpes Simplex Virus Glycoprotein D Bound to the Human Receptor Nectin-1
Source: PLoS Pathog. 2011 Sep 29;7(9):e1002277. doi: 10.1371/journal.ppat.1002277 (PMC3182920; doi:10.1371/journal.ppat.1002277)
Supplement: Table S1 — Data collection and refinement statistics. (DOC) [file ppat.1002277.s004.doc]

|  | | |
| --- | --- | --- |
| *Data collection* | |  |
| Space group | | P3(2)21 |
| Cell dimensions *a*, *b*, *c* (Å) | | 188.00, 188.03, 185.01 |
| Resolution (Å) | | 4.0 (4.1–4.0) ^†^ |
| *R*_sym_ | | 7.6 (27.8) |
| <I/σ> | | 16.5 (4.9) |
| Completeness (%) | | 99.0 (99.9) |
| Redundancy | | 3.6 (3.6) |
| *Refinement Statistics* | |  |
| Resolution (Å) | | 30–4.0 (4.1-4.0) |
| Polypeptide chains | | 6 |
| Unique reflections | | 30,227 (2,244) |
| Protein Atoms | | 9,632 |
| RMSD bond lengths (Å) | | 0.017 |
| RMSD bond angles (deg) | | 1.745 |
| Mean B values (Å^2^) | | 38.0 |
| Residues in the allowed region of the Ramachandran plot (%) | | 98.3 |
| Residues in the most favored region of the Ramachandran plot (%) | | 76.7 |
| *R_work_* | | 26.5 (34.4) |
| *R_free_* | | 28.9 (36.8) |
|  |  | |

^†^ Values in parentheses correspond to the statistics for the highest resolution shell.
